# Rilzabrutinib for the Treatment of Immune Thrombocytopenia

**DOI:** 10.1111/ejh.14425

**Published:** 2025-04-13

**Authors:** Caterina Labanca, Enrica Antonia Martino, Ernesto Vigna, Antonella Bruzzese, Francesco Mendicino, Giulio Caridà, Eugenio Lucia, Virginia Olivito, Veronica Manicardi, Nicola Amodio, Antonino Neri, Fortunato Morabito, Massimo Gentile

**Affiliations:** ^1^ Hematology Unit, Azienda Ospedaliera Annunziata Cosenza Italy; ^2^ Laboratorio di Ricerca Traslazionale, Azienda USL‐IRCSS Reggio Emilia Reggio Emilia Italy; ^3^ Department of Experimental and Clinical Medicine University Magna Graecia of Catanzaro Catanzaro Italy; ^4^ Scientific Directorate, IRCCS of Reggio Emilia Reggio Emilia Italy; ^5^ Gruppo Amici Dell'ematologia Foundation‐GrADE Reggio Emilia Italy; ^6^ Department of Pharmacy, Health and Nutritional Science University of Calabria Rende Italy

**Keywords:** Bruton's tyrosine kinase inhibitor, platelet response, refractory immune thrombocytopenia, rilzabrutinib

## Abstract

Advancements in the understanding of ITP pathogenesis have led to significant improvements in disease management through the use of both traditional immunosuppressive strategies and novel targeted therapies. However, a subset of patients remains refractory to treatment or achieves only transient benefits, underscoring the need for alternative therapeutic approaches. Bruton's tyrosine kinase (BTK) inhibitors have emerged as a promising strategy for autoimmune cytopenias, including ITP, due to their ability to modulate key immune pathways. Rilzabrutinib, an oral, reversible BTK inhibitor, represents a novel therapeutic approach for ITP. Rilzabrutinib, an oral, reversible BTK inhibitor, offers a novel mechanism of action by preserving platelet aggregation while reducing macrophage‐mediated platelet clearance, distinguishing it from irreversible BTK inhibitors. This review provides an updated and comprehensive analysis of the Phase 1/2 LUNA 2 trial and its long‐term extension, contextualizing rilzabrutinib within the broader treatment landscape. We also offer a comparative assessment of other BTK inhibitors investigated for ITP and discuss rilzabrutinib's potential positioning relative to existing therapies, including thrombopoietin receptor agonists (TPO‐RAs), rituximab, fostamatinib, and immunosuppressants. Results from the phase 1/2 LUNA 2 trial and its long‐term extension demonstrated that Rilzabrutinib induced a durable platelet response in 40% of patients, with a median time to response of 11.5 days. The treatment exhibited a favorable safety profile, with predominantly grade 1 or 2 adverse events and no significant safety concerns commonly associated with BTK inhibitors, such as increased bleeding risk, hepatic toxicity, or cardiac arrhythmias. Preliminary data presented at ASH 2024 from the ongoing Phase 3 LUNA 3 trial, a randomized, double‐blind study, further support rilzabrutinib's efficacy and long‐term safety. If confirmed, these findings suggest that rilzabrutinib could represent a valuable therapeutic option for patients with refractory ITP, addressing a critical unmet need and potentially redefining treatment paradigms.

## 1 Background

Immune thrombocytopenia (ITP) is an acquired autoimmune disorder characterized by reduced platelet counts (PCs) due to immune dysregulation [1, 2]. The global prevalence of ITP is estimated to range between 10 and 23 per 100 000 individuals, with a higher incidence in females under 70 years of age [3, 4].

Recent advancements in the immunopathology of ITP have provided deeper insights into its complex pathophysiology, identifying key mechanisms, including T‐cell dysregulation, complement activation, and impaired megakaryopoiesis reviewed in [5]. A comprehensive understanding of these pathways is crucial for the development of targeted therapies that address the multifactorial immune dysfunction underlying the disease.

Platelet destruction in ITP is primarily mediated by autoantibodies targeting glycoproteins (GP) IIb/IIIa and GPIb/IX, leading to Fc gamma receptor (FcγR)‐dependent phagocytosis and complement‐mediated clearance in the spleen and liver [6, 7, 8]. In addition to FcγR‐independent mechanisms, platelet desialylation followed by Ashwell‐Morell receptor (AMR)‐mediated hepatic clearance represents an FcγR‐independent pathway contributing to platelet turnover [7, 8, 9, 10, 11]. Furthermore, in addition to antibody‐driven destruction, cytotoxic CD8+ T cells exacerbate thrombocytopenia by directly targeting platelets and megakaryocytes [12, 13, 14], while regulatory T cell (Treg) deficiency perpetuates autoreactivity, further compromising platelet production [15, 16].

The complement system, particularly the classical pathway, plays a crucial role in platelet opsonization and lysis, bridging humoral immunity to platelet clearance [17, 18, 19, 20, 21, 22]. Beyond peripheral platelet destruction, ITP also involves impaired thrombopoiesis due to both direct immune‐mediated megakaryocyte damage and inadequate thrombopoietin (TPO) levels [23, 24, 25, 26].

This combination of increased platelet clearance and defective production results in persistent thrombocytopenia, which is associated with an elevated risk of spontaneous bleeding, impaired hemostasis, and, in severe cases, life‐threatening hemorrhages [27]. Additionally, ITP significantly impacts quality of life, with symptoms such as psychological distress due to the unpredictable risk of bleeding episodes [28]. Paradoxically, epidemiological studies have reported an increased risk of both venous (VTE) and arterial (ATE) thromboembolism in ITP patients compared to age‐ and gender‐matched controls, suggesting a complex interplay between thrombosis and bleeding in the disease [29].

Given the multifaceted pathophysiology of ITP, the treatment strategies have evolved considerably, incorporating both traditional immunosuppressive approaches and novel targeted therapies.

Table 1 provides a structured overview of the current ITP treatments, categorizing therapies based on their mechanisms of action and role in disease management.

**TABLE 1:** Current treatment strategies for immune thrombocytopenia (ITP) and their mechanisms of action.

Therapy category	Treatment	Mechanism of action
First‐line	Corticosteroids (Prednisone, Dexamethasone)	Broad immunosuppression; inhibits B‐ AND T‐cell activation, reducing antibody production and macrophage‐mediated platelet destruction
	Intravenous Immunoglobulin (IVIg)	Blocks FCγ receptors on macrophages, preventing antibody‐dependent platelet clearance; saturates FcRN accelerating IgG autoantibody degradation
Second‐line therapies	Thrombopoietin‐Receptor Agonists (TPO‐RAS) (Romiplostim, Eltrombopag, Avatrombopag)	Stimulate platelet production by activating megakaryocytes through tpo receptor signaling
	Anti‐CD20 therapy (Rituximab)	Depletes CD20^+^ B cells, reducing autoantibody production and modulating immune dysregulation in ITP
	Splenectomy	Removes the primary site of platelet destruction and autoantibody production in the spleen
Novel and emerging therapies	FcRN Inhibitors (Efgartigimod, Rozanolixizumab)	Blocks neonatal Fc receptor (FCRN) to reduce IGG autoantibody levels, preventing platelet destruction
	Complement inhibitors (sutimlimab)	Inhibits the classical complement pathway (C1S), preventing complement‐mediated platelet opsonization and lysis
	BTK Inhibitor (Rilzabrutinib)s	Inhibits BTK‐mediated B‐cell activation and fcγr signaling in macrophage, reducing antibody‐mediated platelet phagocytosis without impairing platelet function.
	Syk Inhibitors (Fostamatinib)	Inhibits FcγR‐mediated signaling in macrophages, preventing antibody‐dependent platelet destruction
	Neuraminidase Inhibitors (Oseltamivir)	Prevents platelet desialylation and their clearance by hepatocytes, preserving platelet lifespan
T‐cell modulation strategies	Low‐dose IL‐2	Enhances regulatory T‐cell (Treg) function, restoring immune tolerance
	Anti‐CD40/CD154 agents	Disrupt CD40‐CD154 interaction, impairing B‐cell activation and autoantibody production
Other agents Immunosuppressive or immunomodulatory agents	Azathioprine	Immunosuppressive agent that modulates RAC1 to induce T‐cell apoptosis, along with other unknown immunosuppressive mechanisms
	Cyclosporine‐A	Immunomodulatory agent that inhibits cytokine production involved in T‐cell activation, specifically blocking interleukin 2
	Danazol	Increases platelet counts by modulating the immune response and antagonizing estrogen
	Mycophenolate Mofetil	Inhibits T‐ and B‐cell proliferation, reduces cytotoxic T‐cells and antibody production, and prevents lymphocyte and monocyte adhesion to endothelial cells via the glycosylation of cell adhesion molecules

First‐line therapies, including corticosteroids and intravenous immunoglobulin (IVIg), remain the cornerstone of initial treatment, exerting their effects through general immunosuppression or macrophage inhibition [30, 31, 32, 33]. However, their temporary efficacy and associated side effects [30, 31] often necessitate second‐line options. However, their transient efficacy and associated toxicities necessitate the use of second‐line options, such as rituximab, which targets CD20+ B cells to deplete autoantibody‐producing plasma cells. Despite its immunosuppressive effects, the long‐term benefits of rituximab remain uncertain [34]. Splenectomy, once a mainstay of ITP management, has declined in use, but remains an option for refractory cases, offering a durable response in approximately 60%–70% [35, 36, 37]. Despite its efficacy, splenectomy carries risks of surgical complications and long‐term immunosuppression‐associated infections [38].

Thrombopoietin‐receptor agonists (TPO‐RAs) such as romiplostim, eltrombopag, and avatrombopag have significantly shifted the treatment paradigm by stimulating megakaryocyte maturation and platelet production [5, 39, 40]. However, a substantial proportion of patients fail to achieve sustained remission, and concerns regarding rebound thrombocytopenia and thrombotic events upon discontinuation remain.

Additional immunosuppressive and immunomodulatory agents, including azathioprine, cyclosporine A, danazol, dapsone, hydroxychloroquine, mycophenolate mofetil (MMF), and vinca alkaloids, have been utilized in ITP, with response rates ranging from 30%–60% depending on the agent and patient population. However, their use has diminished with the advent of more targeted therapies and is now largely reserved for refractory cases [40]. Over the past two decades, various combination strategies have been explored for both first‐line and subsequent therapies, but none have been widely adopted or endorsed by current guidelines due to inconsistent efficacy and limited consensus [40].

Chronic ITP significantly impacts the quality of life, leading to persistent fatigue and anxiety due to the risk of bleeding. Severe hemorrhages, including intracranial bleeding, become more likely with persistently low platelet counts and advanced age [28]. Given the limitations of current therapies, ongoing research and clinical trials are essential for developing novel treatments that offer greater efficacy, tolerability, and long‐term disease control [41, 42, 43].

In recent years, novel targeted therapies have emerged, expanding treatment options beyond traditional immunosuppression (Table 1). T‐cell‐directed therapies, such as low‐dose IL‐2 and anti‐CD40/CD154 agents, aim to restore immune tolerance rather than merely suppress immune function [5, 40]. FcRn inhibitors reduce pathogenic autoantibody levels, while complement inhibitors prevent immune‐mediated platelet destruction. Spleen tyrosine kinase (Syk) inhibitors, such as fostamatinib [44, 45, 46] disrupt macrophage and B‐cell signaling, offering new immune‐modulating strategies. Fostamatinib demonstrated clinical efficacy in the FIT‐1 (NCT02076399) and FIT‐2 (NCT02076412) clinical trials [44, 45, 46], leading to its approval for ITP treatment in the United States (April 2018) [47] and the European Union (January 2020) [48]. HMPL‐523 has completed its Phase 1b trial in China (NCT03951623).

HMPL‐523, another oral Syk inhibitor, has completed its Phase 1b trial in China (NCT03951623) and is currently under evaluation in a Phase 3 trial (NCT05029635) for refractory ITP [49, 50, 51].

Bruton tyrosine kinase (BTK) inhibitors have recently gained attention as a potential therapeutic class in ITP, given their ability to modulate B‐cell activation, autoantibody production, and FcγR‐mediated platelet destruction [52, 53].

Several BTK inhibitors are in clinical development for ITP, including orelabrutinib, which has shown promising preliminary efficacy in Phase I/II trials [NCT05124028, NCT05020288, NCT05232149] and is currently being evaluated in a Phase III study [NCT06004856]. The initial Phase 2 data demonstrated that in patients with persistent or chronic ITP, a platelet response was achieved in 40% of those receiving a daily dose of 50 mg. Treatment was associated with an improvement in bleeding symptoms, particularly in patients with prior exposure to corticosteroids and/or IVIg. However, the interpretation of these findings is limited by the relatively small sample size [54].

Zanubrutinib, a next‐generation BTKi, exerts its activity by covalently binding to the cysteine residue at position 481 of the BTK protein, thereby preventing its tyrosine phosphorylation at site 223. Ongoing clinical trials are evaluating its efficacy in ITP, both as monotherapy [NCT05279872, NCT05214391] and in combination with high‐dose dexamethasone [NCT05369364] or Eltrombopag [NCT05369377]. Ding et al. conducted a retrospective case series analyzing the efficacy and safety of Zanubrutinib in four patients with relapsed/refractory ITP. Among them, one patient achieved a complete response, while two experienced a partial response. One patient, however, died following treatment discontinuation for an intracranial hemorrhage due to a fall in PCs. No kidney or hepatic toxicities were reported [55].

This review focuses on rilzabrutinib, an oral, reversible Bruton tyrosine kinase (BTK) inhibitor with a distinct mechanism of action that preserves platelet aggregation while reducing macrophage‐mediated platelet clearance [52, 53]. Ongoing clinical trials are evaluating its efficacy and safety relative to existing ITP treatments, underscoring the need for continued research into targeted therapies that improve long‐term disease control while minimizing treatment‐related toxicities.

## 2 Rationale for Using RILZABRUTINIB

Bruton tyrosine kinase (BTK), a cytoplasmic kinase belonging to the Tec family, is widely expressed in B cells and innate immune cells, where it plays a key role in signaling cascades activated by receptors such as the B‐cell receptor (BCR) in B cells and the high‐affinity IgE receptor (FcεRI) in megakaryocytes [56, 57, 58, 59, 60, 61, 62, 63, 64]. Upon activation, BTK undergoes phosphorylation at Y551 by SYK or SRC family kinases (e.g., LYN) followed by autophosphorylation at Y223, enabling interactions with adapter proteins such as SLP‐76 and BLNK. This cascade subsequently activates phospholipase C gamma 2 (PLCγ2), leading to downstream signaling through NF‐κB and NFAT [28, 29, 30, 31, 32, 33]. Additionally, BTK regulates chemokine‐mediated pre‐B cell homing into lymphoid organs, toll‐like receptor (TLR) signaling, and FcγR‐mediated immune response [52, 53, 65, 66].

BTK inhibition disrupts these pathways, leading to reduced autoantibody production and impairing macrophage function, FcγR‐mediated phagocytosis, FcεR‐induced mast cell degranulation, granulocyte migration, and inflammatory mediator release. Expanding knowledge of BTK's immunomodulatory role has driven the development of BTK inhibitors (BTKi) for B‐cell malignancies and autoimmune diseases [67]. Given its capacity to attenuate autoantibody‐mediated platelet destruction while allowing for oral administration, BTK inhibition represents a promising therapeutic strategy for ITP management.

The potential for BTK inhibition in ITP was initially observed in patients with B‐cell malignancies complicated by ITP, in whom initiation of ibrutinib therapy led to high rates of ITP remission [68].

However, current FDA‐approved BTK inhibitors lack selectivity, leading to off‐target inhibition of platelet collagen receptors (GPVI, GPIb) and integrins (αIIbβ3), which may compromise platelet aggregation and increase bleeding risk—a critical concern in thrombocytopenic patients [69, 70].

To overcome these limitations, efforts have focused on developing more selective BTK inhibitors that effectively suppress autoantibody production and phagocyte‐mediated platelet destruction while preserving platelet function to minimize bleeding risk.

**FIGURE 1:**
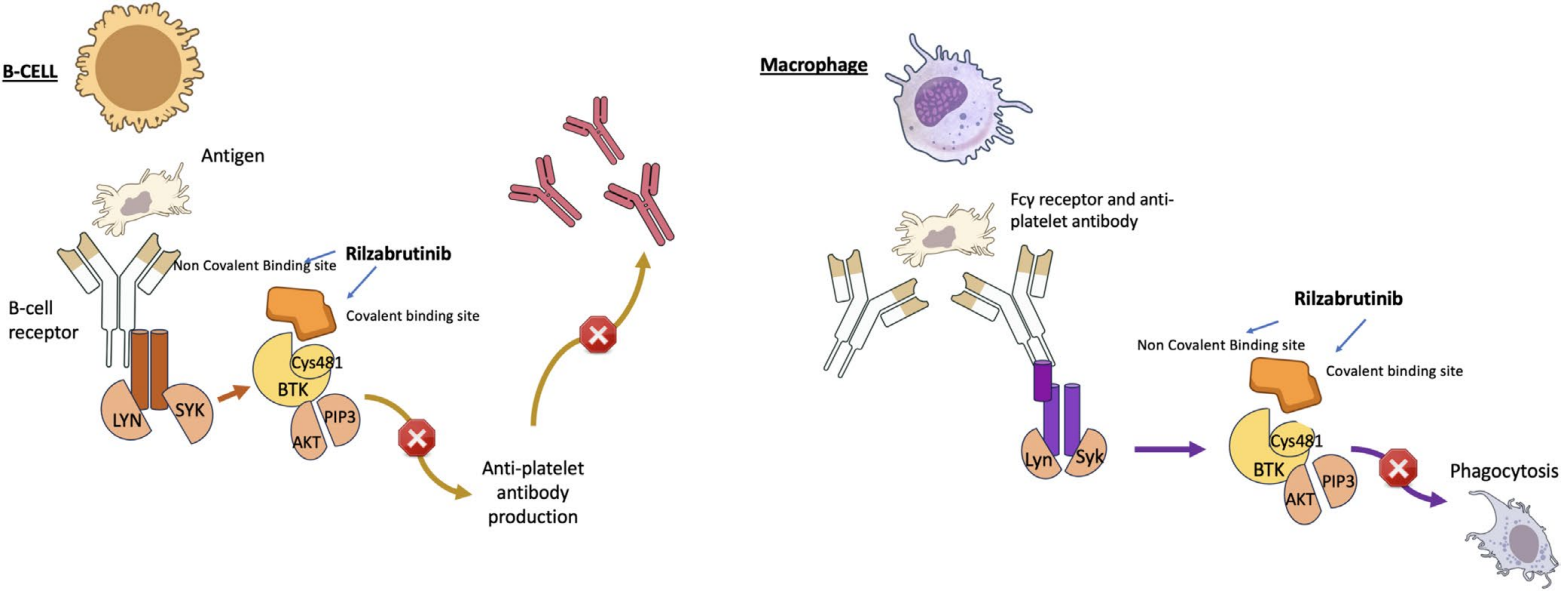
Mechanism of action of rilzabrutinib. Rilzabrutinib, a selective and reversible Bruton tyrosine kinase (BTK) inhibitor, modulates immune responses through two key mechanisms. (Left panel) In B cells, Rilzabrutinib inhibits BTK activation downstream of the B‐cell receptor (BCR), thereby reducing anti‐platelet autoantibody production. (Right panel) In macrophages, Rilzabrutinib blocks Fcγ receptor (FcγR)‐mediated signaling by preventing BTK activation, thereby inhibiting the phagocytosis of antibody‐coated platelets. Unlike irreversible BTK inhibitors, Rilzabrutinib binds non‐covalently to BTK, preserving platelet function and reducing off‐target effects.

Rilzabrutinib is an oral, reversible, and highly selective BTK inhibitor designed for immune‐mediated diseases. It exerts its effects through a dual mechanism: suppressing B‐cell activation and inhibiting FcγR‐mediated phagocytosis of antibody‐coated cells in the spleen and liver [70, 71, 72]. Unlike non‐selective BTKi, rilzabrutinib does not form permanent covalent bonds with target proteins or peptides, allowing for more controlled inhibition and improved safety [70, 72, 73]. Notably, it has been shown to preserve platelet aggregation in both healthy individuals and patients, thereby mitigating the risk of bleeding complications [70, 74] (Figure 1).

A phase I/II open‐label clinical trial demonstrated that rilzabrutinib achieved a rapid and sustained platelet response in patients with refractory ITP, with an overall favorable safety profile characterized by mild adverse events (e.g., diarrhea, nausea, and fatigue) and no reported grade ≥ 2 bleeding or thrombotic events [75, 76]. In recognition of its therapeutic potential, rilzabrutinib was granted orphan drug designation for ITP in the U.S. in October 2018 and received FDA Fast Track Designation on November 18, 2020 [77]. The ongoing phase III LUNA 3 RCT (NCT04562766) is currently evaluating its efficacy and safety in adolescents and adults (≥ 12 years) with persistent or chronic ITP. If confirmed effective, rilzabrutinib could represent a major advancement in ITP treatment by providing targeted immunomodulation while minimizing bleeding risks [78, 79].

## 3 Pharmacokinetics and Pharmacodynamics

Preclinical in vitro and in vivo studies have shown that rilzabrutinib exhibits a favorable pharmacokinetic and pharmacodynamic profile, supporting its therapeutic potential for immune‐mediated diseases [71, 73, 80, 81]. As a reversible, orally administered BTK inhibitor, Rilzabrutinib (PRN1008) has a short half‐life (approximately 3–4 h) but achieves sustained BTK target occupancy (> 90% within 4 h and sustained over 24 h) suggesting a prolonged pharmacodynamic effect despite its transient systemic exposure [70, 71, 72, 73].

Rilzabrutinib uniquely employs a dual binding mechanism, engaging in both reversible covalent and non‐covalent interactions. This selective and reversible covalent interaction at Cys481 enhances specificity while minimizing off‐target effects and avoiding permanent protein modifications. Unlike irreversible BTKi, rilzabrutinib preserves platelet function and does not impair platelet aggregation, thereby reducing the risk of bleeding—a crucial consideration in patients with ITP [71, 73, 80].

Pharmacokinetic evaluations, including phase I trials in healthy participants, have provided key insights into metabolism and potential drug–drug interactions (DDIs). A study by Rask‐Madsen et al. investigated DDIs and found that rilzabrutinib exposure remained unaffected by quinidine (a P‐gp and CYP2D6 inhibitor) but was significantly reduced (~80%) by rifampin (a CYP3A inducer), confirming its metabolism as primarily CYP3A‐mediated [81, 82, 83]. Additionally, a two‐part phase I trial (anzctr.org.au ACTRN12618001036202) by Ucpinar et al. assessed supratherapeutic dosing and cardiac safety. While the 1200 mg dose was discontinued due to gastrointestinal intolerance, rilzabrutinib exhibited no clinically relevant effects on ECG parameters, including QTc interval prolongation [84].

Rilzabrutinib undergoes hepatic metabolism primarily via cytochrome P450 (CYP) 3A, generating inactive metabolites, with minimal renal excretion [85, 86]. Its pharmacokinetic profile supports further investigation across a range of autoimmune and inflammatory conditions.

## 4 Clinical Trials

Initial clinical trials have provided empirical validation of preclinical findings, demonstrating the safety and efficacy of rilzabrutinib in ITP. The ongoing Phase I/II and Phase III trials, summarized in Tables 2 and 3, aim to further characterize their clinical benefits and long‐term safety across different patient populations and disease stages.

**TABLE 2:** Description of phase 1/2 trial LUNA 2.

ClinicalTrials.gov	Population and treatment	Primary outcomes		References
	R/R ITP patients treated with rilzabrutinib (*N*)	Safety	Efficacy	
NCT03395210 (LUNA 2 trial) Active, non‐recruiting Phase 1/2	*Part A* (*N = 60*) 200 mg/day 400 mg/day 300 mg 2/day 400 mg 2/day	*Treatment‐related AEs* Any AEs 52% [G1 45%; G2 25%] Diarrhea 32% [G1 27%; G2 5%] Nausea 30% [G1 27%; G2 3%]Fatigue 10% [G1 8%; G2 2%]	*Primary platelet response [% n/N] 40% (24/60)* 4/24 concomitant TPO‐RAs 8/24 concomitant glucocorticoids 3/24 concomitant TPO‐RAs and glucocorticoids 9/24 no ITP concomitant med.	Kuter DJ et al. [45]
	*Part B* (*N = 26*) 400 mg 2/day	*Treatment‐related AEs* ‐ Any AEs 62% [G1 54%; G2 15%; G3 4%]—Diarrhea 35% [G1 27%; G2 8%]—Headache 23% [G1 19%; G2 4%]—Nausea 15% [G1 15%]—Fatigue 0	*Durable PCs response [% n/N] 35% (9/26)*	Cooper N et al. [46]
	*LTE* (*N = 16*) 400 mg 2/day	*Treatment‐related AEs* ‐ Any AEs (≥ 1 AEs) 19% [G1 13%; G2 13%]—Upper respiratory tract infection 6% [G2 6%]—Rhinorrhea 6% [G2 6%]—Vulvovaginal dryness 6% [G2 6%] ‐ Cough 6% [G1 6%] ‐ Diarrhea 6% [G1 6%]	*Median weeks PCs increased ≥ 20 × 10* ^*9*^ */L above baseline* – Rilzabrutinib mono: 3 mo: 11 w; 6 mo 21 w – Rilzabrutinib + ITP concomitant med: 3 mo 13 w; 6 mo 21 w *Median weeks PCs ≥ 30 × 10* ^*9*^ */L* – Rilzabrutinib mono: 3 mo: 12 w; 6 mo 21 w – Rilzabrutinib + ITP concomitant med: 3 mo 13 w; 6 mo 21 w *Median weeks PCs ≥ 50 × 10* ^*9*^ */L* – Rilzabrutinib mono: 3 mo: 11 w; 6 mo 20 w – Rilzabrutinib + ITP concomitant med: 3 mo 13 w; 6 mo 19 w	Kuter DJ et al. [54]

Abbreviations: AEs, adverse events; G1, grade 1; G2, grade 2; LTE, Long Term extension; med, medication; mo, months; mono, monotherapy; *N*, number of all patients; n/N, patients/all patients; PCs, platelet counts; R/R ITP patients, patients with immune thrombocytopenia who are refractory or relapsed; TPO‐RAs, thrombopoietin receptor agonists; w, weeks.

**TABLE 3:** Study design of Phase 3 trial LUNA.

ClinicalTrials.gov	Population	Treatment	Primary endpoints	Secondary endpoints
NCT04562766 (LUNA 3 trial) Active, non‐recruiting Phase 3 Kuter et al. [55]	Adults (*N* = 194) and adolescent (*N* = 30) patients with persistent or chronic ITP	Rilzabrutinib 400 mg bid vs. Placebo	*Durable platelet response*: “PCs ≥ 50 × 10^9^/L for ≥ two‐thirds of at least 8 non‐missing weekly scheduled platelet measurements during the last 12 weeks of the 24‐week blinded treatment period in the absence of rescue therapy.”	– Frequency and severity of TEAEs – Frequency and severity of bleeding TEAEs – Plasma concentrations of rilzabrutinib – Change from baseline on the Symptoms, Bother, and Activity domains of the ITP‐PAQ in adult patients (≥ 18 years) – Change from baseline in disease‐specific QoL as measured by the Kids' ITP Tools (ITP‐KIT) score in pediatric participants.

Abbreviations: bid, twice daily; ITP, Immune thrombocytopenia; ITP‐KIT, Kids' ITP Tools; ITP‐PAQ, ITP Patient Assessment Questionnaire; *N*, number of all patients; PCs, platelet counts; QoL, quality of life; TEAEs, Treatment‐Emergent Adverse Events; vs., versus.

### 4.1 Phase I/II Trial (NCT03395210)

Kuter et al. reported results from part A of an international, two‐part (Part A and B) adaptive, open‐label, dose‐finding, Phase I/II clinical trial (NCT03395210), designed to assess the safety and efficacy of rilzabrutinib in previously treated patients with ITP [75]. This trial enrolled 60 patients with a median baseline PC of 15 × 10^9^/L, a median disease duration of 6.3 years, and a median of four previous treatments (range, 1–17). The most common previous therapies included glucocorticoids (92%), TPO‐RAs (58%), IVIg (43%), and rituximab (40%); 25% of the patients had undergone splenectomy.

To allow for a more comprehensive evaluation of rilzabrutinib, concomitant stable‐dose ITP therapies (e.g., corticosteroids or TPO‐Ras) were permitted. Patients were treated using an intra‐patient dose‐escalation strategy over 24 weeks, with initial dosing regimens of 200 mg once daily (the lowest dose), 400 mg once daily, 300 mg twice daily, and 400 mg twice daily. The primary endpoints were safety and platelet response, with response defined as two consecutive PCs ≥ 50 × 10^9^/L and an increase of ≥ 20 × 10^9^/L from baseline without the rescue therapy [75].

Efficacy data from Part A showed that 40% (24/60) of patients achieved the primary platelet response with a median time to response of 167.5 days. Among patients who started treatment at the highest dose, the response rate was also 40% (18/45). The median time to first platelet response (PCs > 50 × 10^9^/L) was 11.5 days, and responders maintained PCs ≥ 50 × 10^9^/L for a mean of 65% of weeks. Subgroup analyses demonstrated consistent response rates across patients with ≥ 4 prior therapies (36%), those not receiving concurrent ITP treatment (45%), and splenectomized patients (33%). Of the 24 responders, four patients received only TPO‐RAs, eight received only glucocorticoids, and four received both. Safety analysis indicated that rilzabrutinib was well‐tolerated, with no unexpected safety signals observed [75].

Based on these findings, the 400 mg twice‐daily (BID) regimen was selected for Part B. Cooper et al. published the efficacy and safety data from this phase, in which 26 patients were treated with rilzabrutinib at 400 mg BID for 24 weeks, with an option to enter a long‐term extension (LTE) phase.

Part B enrolled patients with a median baseline PC of 13 × 10^9^/L, a median disease duration of 10.3 years, and a history of six prior ITP therapies. Notably, 46% of the cohort had undergone splenectomy. The primary endpoint—a durable platelet response (PCs ≥ 50 × 10^9^/L in ≥ 67% of study weeks during the last 12 weeks of treatment)—was achieved in 35% (9/26) of patients, with a median time to response of 8 days. In contrast, 65% (17/26) of non‐responding patients exhibited persistently low PCs across all assessments up to day 148. Subgroup analyses showed consistent efficacy across baseline characteristics, prior treatments, and concomitant therapies. The safety profile remained comparable to Part A, further supporting rilzabrutinib's tolerability [76].

The LTE phase of the NCT03395210 trial evaluated the durability of response and long‐term safety of rilzabrutinib at 400 mg BID in 16 patients who responded in the initial study phases. These patients had a median ITP duration of 4.3 years (range, 0.5–18.4) and had received a median of three prior therapies (range, 1–9); 19% had undergone splenectomy. Baseline median PC was 87 × 10^9^/L, with values stratified by treatment: 68 × 109/L 10^9^/L for those on rilzabrutinib monotherapy (*n* = 5), and 156 × 10^9^/L for those on concomitant TPO‐RAs and/or corticosteroids (*n* = 11).

After a median treatment duration of 478 days, 69% (11/16) of patients remained on rilzabrutinib, with 93% maintaining PCs of ≥ 50 × 10^9^/L in over half of their monthly assessments. The median percentage of LTE weeks with PCs ≥ 30 × 109/L was 100%, while for PCs ≥ 50 × 10^9^/L, it was 80%. Notably, five patients transitioned from combination therapy to rilzabrutinib monotherapy, maintaining a median PC of 106 × 10^9^/L for approximately 3–6 months after discontinuing their concomitant ITP treatments [86]. These findings further support the long‐term efficacy and tolerability of rilzabrutinib in ITP.

Building upon these promising results, a Phase III randomized double‐blind LUNA 3 trial (NCT04562766) was initiated to evaluate rilzabrutinib in adults and adolescents (≥ 12 years) with persistent or chronic ITP. LUNA 3 represents one of the largest clinical trials conducted in patients with primary ITP to date, uniquely designed to include both adult (*n* = 194) and adolescent (*n* = 30) participants [78].

Eligible patients had baseline PCs of < 30 × 10^9^/L (with no single PCs exceeding 35 × 10^9^/L) based on two assessments conducted at least 5 days apart within the 14 days preceding treatment initiation. Participants were randomized to receive rilzabrutinib 400 mg BID or placebo for up to 60 weeks, including a 4‐week screening period, 12 to 24 weeks of blinded treatment, a 28‐week open‐label period, and a four‐week follow‐up. An LTE period extends up to 12 months for adults and pediatric patients.

The primary endpoint of the trial is durable platelet response (PCs ≥ 50 × 10^9/^L in ≥ 67% of at least eight weekly assessments during the final 12 treatment weeks). This endpoint differs from the criteria established by the International Working Group consensus, which defines a response as achieving a PCs between 30 × 10^9^/L and 100 × 10^9^/L with at least a twofold increase from the baseline count, while a complete response is defined as PCs ≥ 100 × 10^9^/L, both in the absence of bleeding [4].

Secondary endpoints include safety, bleeding reduction, quality of life (assessed using ITP‐PAQ for adults and Kids ITP Tools for pediatric patients), and rescue therapy use. Adverse events are evaluated per the modified Common Terminology Criteria for Adverse Events (CTCAE) version 5.0 criteria [87, 88].

Interim results from LUNA 3, reported at ASH 2024, included data from 202 adult patients (rilzabrutinib: *n* = 133; placebo: *n* = 69) with median ITP durations of 8.1 years and 6.2 years, respectively. Platelet response (PC ≥ 50 × 10^9^/L) was achieved in 65% (86/133) of rilzabrutinib‐treated patients versus 33% (23/69) in the placebo group. Notably, durable response—the primary outcome—was observed in 23% (31/133) of rilzabrutinib patients and 0% of placebo patients.

The median time to PC ≥ 50 × 10^9^/L or ≥ 30–< 50 × 10^9^/L with a ≥ 2‐fold increase from baseline was 36 days in the rilzabrutinib group but was not achieved in the placebo group. Additionally, rilzabrutinib reduced rescue therapy use by 52%. Quality‐of‐life analyses demonstrated improvements in fatigue (ITP‐PAQ item 10, mean change at week 13: +7.95 vs. −0.13) and bleeding (IBLS score at week 25: −0.04 vs. +0.05) [79].

These findings reinforce rilzabrutinib's potential to provide targeted, durable platelet responses while preserving safety and quality of life in patients with ITP.

## 5 Safety

The safety data from the phase I/II LUNA 2 trial and its LTE study have demonstrated a favorable safety profile for rilzabrutinib in patients with persistent or chronic ITP.

In Part A of the trial, treatment‐related adverse events (TrAEs) were reported in 52% of patients (31/60), all of which were classified as grade 1 or grade 2 and fully reversible. The most frequently reported TrAES included gastrointestinal symptoms, such as diarrhea (32%) and nausea (30%), followed by fatigue (10%). One case of treatment‐related grade 1 bleeding (contusion) and one case of grade 2 infection (erysipelas) were documented, both of which were resolved with appropriate management.

Importantly, rilzabrutinib did not exhibit safety concerns typically associated with Bruton's tyrosine kinase (BTK) inhibitors, including infections, liver toxicity, or cardiac arrhythmias. A single patient death was reported, deemed unrelated to rilzabrutinib treatment, as the patient had discontinued therapy on day 8 due to exacerbation of pre‐existing Evans syndrome and subsequently died over 4 months later. Rescue medication was required for seven patients (12%), with four discontinuing rilzabrutinib as a result. The need for rescue therapy was primarily observed among those receiving lower rilzabrutinib doses (200 mg once daily, 300 mg twice daily, and 400 mg twice daily). Notably, an evaluation of bleeding risk using the ITP‐specific bleeding assessment tool indicated no increase in bleeding events from baseline to the end of the treatment period [75].

In Part B of the trial, AEs of any cause were reported in 85% of patients (22/26), with no deaths recorded. TrAEs were observed in 62% of patients (16/26), the majority being grade 1, including diarrhea (35%), headache (23%), and nausea (15%). A single grade 3 AE was documented, involving elevated serum creatinine phosphokinase levels on day 29. No treatment‐related thrombotic/bleeding event of grade ≥ 2, nor any significant treatment‐related infections were observed.

Treatment‐related SAEs were recorded in four patients, comprising grade 4 thrombocytopenia, grade 3 subcutaneous abscess, grade 3 post‐procedural hemorrhage, and grade 3 syncope; however, investigators determined that none were related to rilzabrutinib, and all resolved.

Bleeding events, assessed using the ITP Bleeding Assessment Tool (ITP‐BAT), were registered in four patients (15%), including cases of grade 2 epistaxis, contusion, hematuria, and a single case of grade 3 post‐procedural hemorrhage. Infection‐related AEs were noted in 50% of patients (13/26), with only one case classified as a serious grade 3 AE (subcutaneous abscess). Exploratory biomarker analyses revealed that median haptoglobin levels increased from 0.75 g/L at baseline to 1.1 g/L at week 25, while immunoglobulin G (IgG) levels decreased from 10.9 to 9.2 g/L (median change −1.55 g/L). No significant changes were recorded in IgG1, IgG4, IgM, or IgE levels. Additionally, TPO levels decreased from baseline to week 25, suggesting a potential modulation of the ITP disease mechanism [76].

The LTE study confirmed the safety findings observed in the Phase 1/2 trial [67]. AEs of any cause were reported in 81% of patients (13/16), with 19% (3/13) experiencing grade ≥ 3 AEs. TrAEs, observed in 19% of patients (3/16), were limited to grade 1 or grade 2 and resolved without complications. These included grade 2 upper respiratory tract infection, rhinorrhea, vulvovaginal dryness, grade 1 diarrhea, and cough. Infections were documented in 25% of patients (4/16), with one patient experiencing a grade 2 upper respiratory tract infection deemed treatment‐related. Three cases of COVID‐19 infection (one grade 2, one grade 3, and one grade 4 pneumonia) were determined to be unrelated to rilzabrutinib. SAEs were reported in three patients (19%), including grade 4 COVID‐19 pneumonia, grade 4 thrombocytopenia, and a combination of grade 3 pneumonia and grade 3 pulmonary embolism. All events were transient and investigator‐determined unrelated to treatment. One patient temporarily interrupted rilzabrutinib for 7 days; another permanently discontinued the treatment, and the third developed AEs 7 days after discontinuation. Liver function and ECG measurement parameters remained stable throughout the LTE period. One patient with a history of hepatic steatosis experienced an elevation in alanine aminotransferase levels (3.4 times the upper normal limit), which resolved within 14 days without treatment discontinuation. Bleeding events, although reported in six patients, were not considered treatment‐related. These included recurrent epistaxis, contusions, gingival bleeding, blood blister formation, cerebral microhemorrhage, and a traumatic hematoma.

Importantly, the disease‐specific bleeding assessment tool showed a decrease in the average bleeding score at 3 months during the LTE period, suggesting an overall reduction in bleeding risk [86].

Collectively, the safety data from the LUNA 2 Phase I/II trial and its LTE extension indicate that rilzabrutinib maintains a well‐tolerated safety profile, with AEs primarily limited to grade 1 or grade 2 severity. Importantly, rilzabrutinib does not appear to be associated with the increased risk of infections, hepatic toxicity, or cardiac arrhythmias observed with other BTK inhibitors. While some bleeding events occurred, they were not linked to rilzabrutinib treatment. These findings the continued clinical development of rilzabrutinib, including the ongoing phase 3 LUNA 3 trial, to further evaluate its long‐term safety and efficacy in patients with ITP.

The first safety data derived from the LUNA 3 trial were reported at ASH 2024, confirming findings from the Phase 1/2 study. The incidence of all‐cause and serious AEs was comparable between rilzabrutinib and placebo groups. Grade > 2 GI AEs occurred at similar rates (6% vs. 4%). The most frequently reported AEs for rilzabrutinib were diarrhea (23% vs. 4%), nausea (17% vs. 6%), headache (8% vs. 1%), and abdominal pain (6% vs. 1%), all of which were grade 1 or 2. Other related AEs were reported in fewer than 5% of patients. One patient with multiple risk factors experienced a grade 3 peripheral embolism (SAE) following rilzabrutinib treatment, while another patient died of pneumonia, t an event deemed unrelated to treatment [79].

## 6 Discussion

Refractory ITP remains a complex and challenging condition, with a subset of patients failing to achieve or sustain responses to existing therapies. This represents a significant unmet clinical need. Current treatment options with established efficacy include thrombopoietic agents, rituximab, and fostamatinib. The advent of TPO‐RAs, such as romiplostim, eltrombopag, and avatrombopag has substantially altered the treatment paradigm of chronic ITP (cITP). By targeting distinct pathogenetic mechanisms, these agents have enabled a shift away from traditional immunosuppressive strategies. However, despite these advances, some patients fail to respond or derive only transient benefits, necessitating prolonged corticosteroid use, frequent IVIG infusions, experimental combined regimens, or off‐label immunosuppressant that are often suboptimal and lack standardization [30, 31, 32, 33, 39].

Advances in the understanding of ITP pathophysiology have facilitated the development of novel targeted therapies with BTKi, particularly rilzabrutinib, which has emerged as a promising candidate. Rilzabrutinib modulates key pathogenic pathways in ITP by inhibiting FcR‐mediated platelet destruction, reducing aberrant antibody production against platelets, and suppressing autoreactive B‐cell activity, thereby lowering antiplatelet antibody levels [70].

A distinguishing characteristic of rilzabrutinib compared to other BTKi is its dual covalent and non‐covalent binding properties, which confer high selectivity and reversible inhibition of BTK without permanently modifying proteins. This feature minimizes the risk of platelet aggregation interference, a known adverse effect associated with other BTKi, and a potential contributor to increased bleeding risk [71, 73, 80]. In addition, rilzabrutinib offers practical advantages, including oral administration, a favorable pharmacokinetic profile, minimal drug–drug interactions, and a reassuring cardiac safety profile, as demonstrated in early trials conducted in healthy volunteers [81, 82, 83, 84, 85, 86].

Results from the Phase I/II LUNA 2 trial, including the LTE, suggest that rilzabrutinib is effective, well tolerated, and safe for patients with ITP who have failed prior treatments. In this trial, 40% of patients met the primary efficacy endpoint, defined as achieving two consecutive PCs ≥ 50 × 109/L and an increase of at least 20 × 109/L from baseline without requiring rescue medication. The median time to response was 11.5 days.

From a safety perspective, rilzabrutinib was well tolerated, with TrAEs primarily limited to grade 1 or 2 severity and no significant grade ≥ 2 bleeding or thrombotic complications. Notably, patients enrolled in the trial were permitted to continue concomitant ITP treatments, such as corticosteroids or TPO‐RAs. Among the 45 patients who reached the primary endpoint, 18 were receiving concurrent therapy, suggesting a potential strategy for optimizing treatment in refractory cases. Additionally, health‐related quality‐of‐life assessments revealed significant and clinically meaningful improvements.

Despite these encouraging findings, the study had limitations, including its open‐label design, lack of a control group, and relatively small sample size [81, 86].

To address these limitations, the ongoing LUNA 3 Phase III trial, was designed as a randomized, double‐blind study comparing rilzabrutinib with placebo in adult and adolescent patients. A key feature of LUNA 3 is its primary endpoint, ‘durable response’, which differs from the criteria established by the International Working Group consensus. In this study, a durable response is defined based on the proportion of available PCs ≥ 50 × 109/L over time, rather than using a fixed threshold of ≥ 30 × 109/L and a doubling of baseline counts at 6 months. This alternative definition accounts for missed platelet assessments and may provide a more comprehensive evaluation of sustained efficacy.

Secondary endpoints of the LUNA 3 trial include safety assessments and quality‐of‐life evaluations. These assessments incorporate disease‐specific and general health‐related quality‐of‐life measures for both adult and pediatric populations, reflecting a growing emphasis on patient‐centered outcomes in ITP management [78, 79].

Currently, no standardized treatment algorithm exists for patients with relapsed or refractory ITP, and management decisions remain highly individualized, based on the patient's age, clinical characteristics, comorbidities, disease duration, and bleeding risk [89, 90].

Despite recent therapeutic advancements, a subset of patients remains refractory to all available options, highlighting the need for additional treatments. Rilzabrutinib represents a novel approach to ITP management, offering a distinct mechanism of action compared to the existing therapies.

While preliminary data from early‐phase studies are promising, the results of the LUNA 3 trial will be pivotal in determining its place in the treatment paradigm.

In conclusion, if rilzabrutinib demonstrates durable efficacy with a manageable safety profile, it may become a first‐in‐class option for patients with refractory ITP, potentially shifting treatment approaches towards earlier integration of targeted therapies. However, further studies will be needed to explore long‐term outcomes, combination strategies, and predictive biomarkers that could optimize patient selection and improve therapeutic success.

## Author Contributions

All authors contributed to the manuscript and were involved in revisions and proofreading. All authors approved the submitted version.

## Conflicts of Interest

The authors declare no conflicts of interest.

## Data Availability

The authors have nothing to report.
